# Management of type 2 diabetes with oral semaglutide: Practical guidance for pharmacists

**DOI:** 10.1093/ajhp/zxaa413

**Published:** 2020-12-23

**Authors:** Michael P Kane, Curtis L Triplitt, Carolina D Solis-Herrera

**Affiliations:** 1 Albany College of Pharmacy and Health Sciences, Albany, NY, USA; 2 University of Texas Health Science Center, San Antonio, TX, USA

**Keywords:** glucagon-like peptides, glucagon-like peptide 1, diabetes mellitus, type 2, patient care, patient care management, pharmacists, pharmacy

## Abstract

**Purpose:**

To provide pharmacists with information on counseling patients with type 2 diabetes (T2D) receiving oral semaglutide.

**Summary:**

Oral semaglutide, the first oral glucagon-like peptide 1 (GLP-1) receptor agonist (GLP-1RA), was approved for the treatment of adults with T2D by the US Food and Drug Administration in September 2019. Semaglutide has been coformulated with the absorption enhancer sodium *N*-(8-[2-hydroxybenzoyl] amino) caprylate to improve bioavailability of semaglutide following oral administration. Oral semaglutide has been shown to have efficacy and safety profiles similar to those of other GLP-1RAs. Many patients with T2D have a complex oral medication regimen to manage their T2D and concomitant chronic comorbid conditions. Therefore, it is important that patients follow the dose administration instructions closely: oral semaglutide should be taken on an empty stomach upon waking with a sip (≤120 mL) of plain water and at least 30 minutes before the first food, beverage, or other oral medications of the day. The most common adverse effects of oral semaglutide are gastrointestinal (typically nausea, diarrhea, and vomiting). It is important for pharmacists to counsel patients prescribed oral semaglutide about optimal oral dosing, why correct dosing conditions are necessary, expected therapeutic response, and effective strategies to mitigate potential gastrointestinal adverse events.

**Conclusion:**

Information and practical strategies provided by pharmacists may facilitate initiation and maintenance of oral semaglutide therapy and ensure that each patient achieves an optimal therapeutic response.

**KEY POINTS**

- Oral semaglutide is the first oral glucagon-like peptide 1 receptor agonist (GLP-1RA) approved by the US Food and Drug Administration for the treatment of type 2 diabetes.
- Oral semaglutide provides patients with a new, potentially more convenient treatment option that offers similar or improved efficacy relative to injectable GLP-1RAs and tolerability consistent with that of the GLP-1RA class.
- Patients treated with oral semaglutide should be counseled about optimal dosing conditions and why they are necessary, the expected therapeutic response, and effective strategies to mitigate potential gastrointestinal adverse events.

Since 2005, glucagon-like peptide 1 (GLP-1) receptor agonists (GLP-1RAs) have become well-established therapies for the treatment of type 2 diabetes (T2D).^1,2^ GLP-1RAs are recognized as an efficacious treatment option with a well-characterized safety profile, offering effective glycemic control, weight loss, and a low risk of hypoglycemia.^3,4^ Both the American Diabetes Association (ADA) guidelines and American Association of Clinical Endocrinologists/American College of Endocrinology (AACE/ACE) consensus statement point to the use of a GLP-1RA as a second-line treatment option in patients with inadequate glycemic control despite use of metformin.^5,6^ Furthermore, AACE/ACE guidance suggests GLP-1RAs as the preferred option, closely followed by a sodium/glucose cotransporter 2 (SGLT2) inhibitor, over other treatment options in this setting.^6^ In certain circumstances, for example if metformin is not tolerated or is contraindicated, some GLP-1RAs may be recommended for first-line therapy instead of metformin.^6^ Independent of glycemic control, both the ADA and AACE/ACE guidelines recommend GLP-1RAs or SGLT2 inhibitors with proven cardiovascular (CV) benefit or efficacy in patients with established atherosclerotic CV disease (ASCVD) or at high CV risk.^5,6^ The AACE/ACE recommendation of an SGLT2 inhibitor or a long-acting GLP-1RA also encompasses patients with heart failure (HF) or chronic kidney disease (CKD).^6^ If an SGLT2 inhibitor with demonstrated CV disease benefits is not tolerated or is contraindicated in patients with HF or CKD, the ADA guidelines recommend a GLP-1RA with demonstrated CV disease benefits.^5^

Until September 2019, 6 GLP-1RA formulations were available for administration by subcutaneous injection, with different administration frequencies (once daily, twice daily, or once weekly).^1,7-11^ However, some patients may prefer oral over injectable medications,^5,12^ and lower treatment adherence has been reported when patients perceive a treatment as difficult or inconvenient.^12^ Oral semaglutide, the first GLP-1RA developed for oral administration, was approved by the US Food and Drug Administration (FDA) in September 2019 for the treatment of adults with T2D.^13^ Pharmacists play a key role in the management and counseling of patients to optimize management of T2D,^14,15^ so it is important that they be aware of the information needed to advise patients and help ensure optimal therapeutic responses are achieved while actively minimizing adverse effects. Therefore, this article focuses on providing pharmacists with practical information about oral semaglutide, including guidance for counseling patients during initiation, titration, and continuation of oral semaglutide treatment.

## Oral delivery of semaglutide

**Figure 1.**
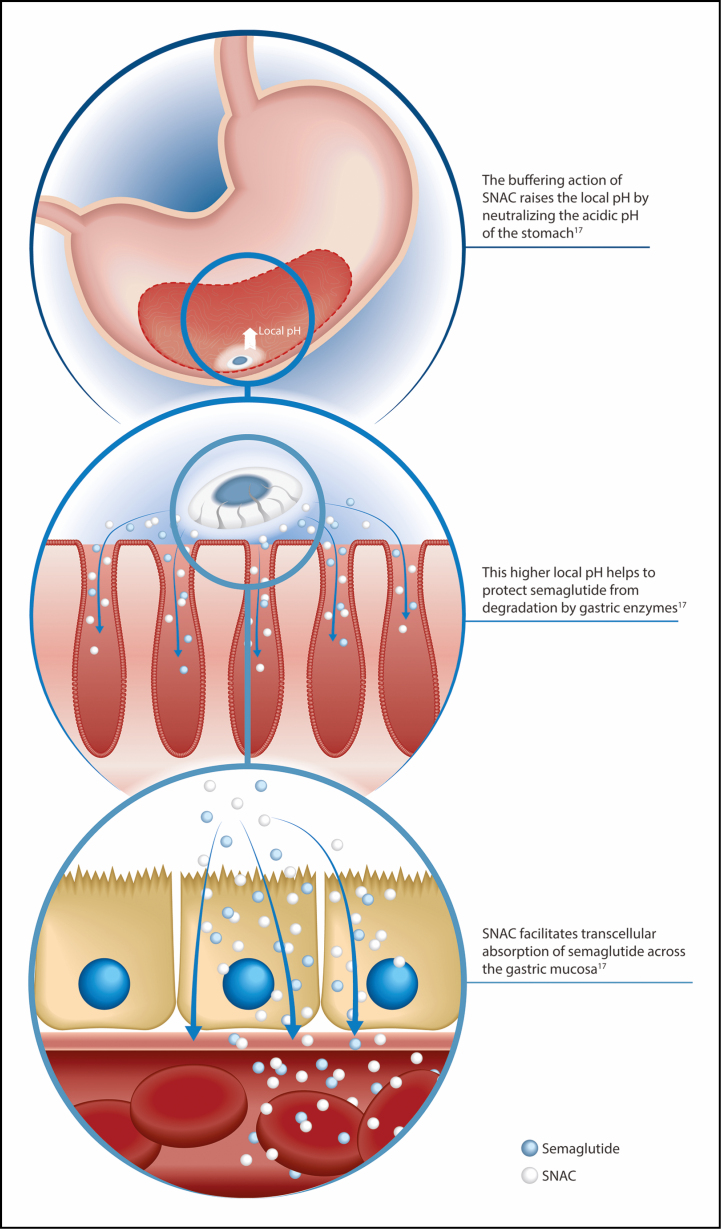
Mechanism of absorption of oral semaglutide. SNAC indicates sodium *N*-(8-[2-hydroxybenzoyl] amino) caprylate.

Oral delivery of peptides, such as semaglutide, can pose a challenge due to enzymatic and acidic degradation of proteins and peptides in the gastrointestinal (GI) tract and the limited permeability of these compounds across the GI epithelium.^16^ To improve bioavailability of semaglutide following oral administration, it has been coformulated with the absorption enhancer sodium *N*-(8-[2-hydroxybenzoyl] amino) caprylate (SNAC) 300 mg.^17,18^ The buffering action of SNAC raises the local pH where the tablet lies in the stomach, which protects semaglutide against proteolytic degradation and denaturation by the highly acidic environment (Figure 1).^17^ SNAC also facilitates transcellular absorption of semaglutide across the gastric mucosa (Figure 1).^17^ Semaglutide is a long-acting GLP-1RA with a half-life (*t*_½_) of approximately 1 week.^19^ Consequently, subcutaneous semaglutide is administered once weekly.^9,19^ Although the *t*_½_ of oral semaglutide is also around 1 week, once-daily administration of oral semaglutide is needed to achieve therapeutic steady-state activity and to mitigate the low bioavailability and high intraindividual variability in exposure seen after a single oral dose.^17,18^ The day-to-day variation in oral semaglutide exposure with once-daily dosing is reduced after steady state is reached in 4 to 5 weeks.^13,18^

## Administration guidance for oral semaglutide

**Figure 2.**
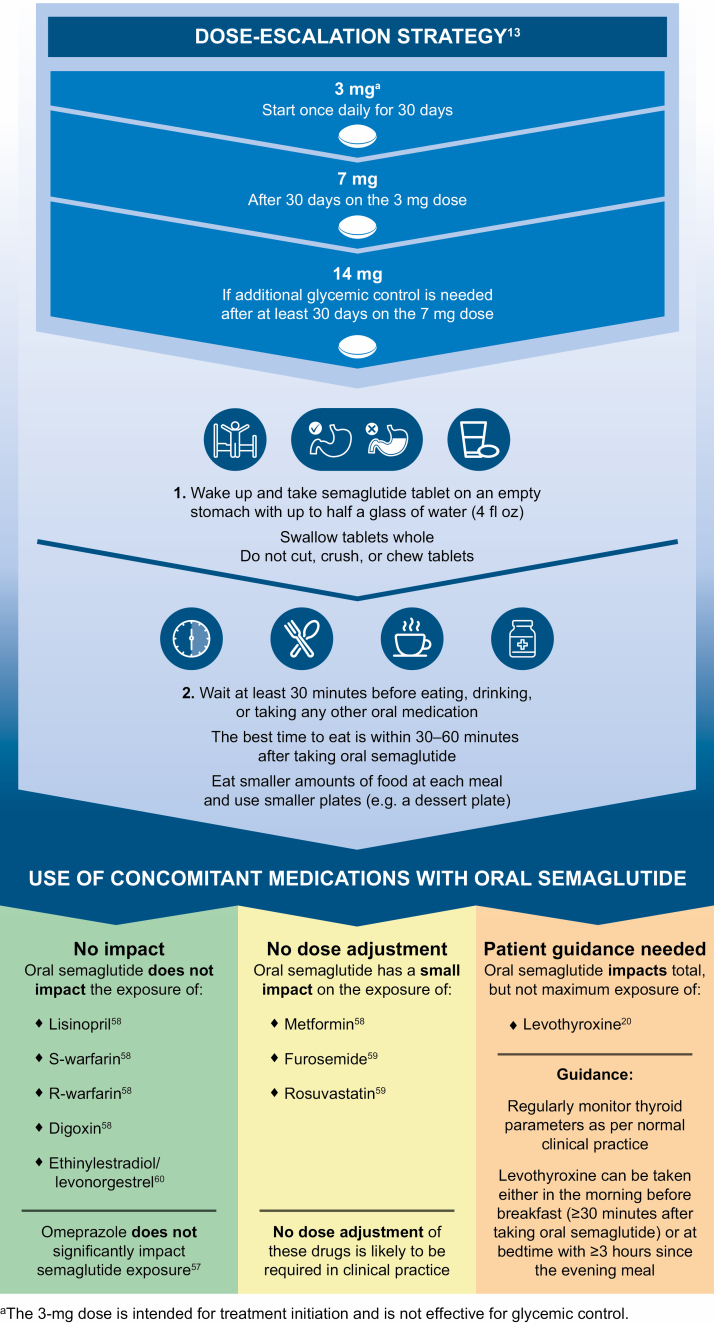
Optimal dosing and administration instructions for oral semaglutide.

The presence of food or multiple tablets in the stomach may affect absorption of oral semaglutide.^17,20^ Early trials demonstrated that after an overnight fast (6-10 hours), oral semaglutide absorption in the stomach was hindered by the presence of food consumed within 30 minutes of dosing.^17,21^ Clinically relevant semaglutide exposure was achieved when oral semaglutide was administered with up to 120 mL of water followed by a postdose fasting period of at least 30 minutes.^21^ Although systemic exposure and time to maximum concentration (*t*_max_) of oral semaglutide increased with longer postdose fasting, no significant difference was identified between postfasting times of 30 and 60 minutes.^21^ In light of the above, it is possible that consuming more than 120 mL (4 fluid ounces) when taking oral semaglutide could adversely affect absorption.^17^ Therefore, patients should be instructed to take oral semaglutide once daily when they first wake up with a sip (up to 120 mL) of plain water.^13^ The tablet should be swallowed whole (and not chewed, split, or crushed). The patient should then wait at least 30 minutes before consuming any food, other drinks, or other oral medications. Oral semaglutide works best if the patient waits 30 to 60 minutes before eating (Figure 2).^13^ Patients should consume smaller quantities of food at each meal to avoid GI adverse events (AEs) such as vomiting.

## Dose escalation guidance for oral semaglutide

A phase 2 trial initially compared the effects of once-daily oral semaglutide (2.5, 5, 10, 20, and 40 mg) and placebo effects on glycemic control in patients with T2D.^22^ A standard dose-escalation strategy was implemented for patients receiving oral semaglutide 5 to 40 mg, with all patients started on doses of 2.5 or 5 mg, which were then doubled every 4 weeks until the randomization dose (5, 10, 20, or 40 mg) was achieved. Additional treatment arms evaluated the effect of dose escalations occurring every 2 weeks (the fast escalation group) or 8 weeks (the slow escalation group), up to 40 mg. At week 26, the mean change in glycated hemoglobin (HbA_1c_) concentration from baseline with oral semaglutide treatment was dose dependent, ranging from –0.7% (at a dose of 2.5 mg) to –1.9% (at a dose of 40 mg) after the 4-week dose escalation schedule. These reductions were significant compared to reductions with placebo use, with estimated treatment differences (ETDs) for oral semaglutide vs placebo ranging from –0.4% to –1.6% (*P* ≤ 0.01 for the 2.5-mg dose, *P* < 0.001 for all other doses). The proportion of patients receiving oral semaglutide and reporting GI AEs increased with higher doses (2.5 mg, 31%; 5 mg, 31%; 10 mg, 54%; 20 mg, 56%; and 40 mg, 61%). In addition, among patients receiving oral semaglutide 40 mg, GI AEs occurred in a higher percentage of patients in the fast escalation group (77%) vs the regular (61%) and slow (54%) escalation groups.

Based on these findings, 3 dose levels of oral semaglutide (3, 7, and 14 mg) were studied in the phase 3a PIONEER trial program. These trials established the efficacy and safety profile of oral semaglutide, and the 3-, 7-, and 14-mg doses were subsequently approved in the United States for the treatment of patients with T2D.^13^ The 3-mg dose of oral semaglutide is intended not as a therapeutic dose but rather as a starting dose to mitigate potential GI AEs.^13^ The 7- and 14-mg doses of oral semaglutide are for use as maintenance doses. To initiate patients on oral semaglutide, it is recommended that a dose-escalation strategy be used, starting with 3 mg once daily for 30 days, then increasing to 7 mg once daily. Following at least 30 days at a dosage of 7 mg once daily, the dose can be further increased to 14 mg if additional glycemic control is required (Figure 2).^13^

## Switching between oral semaglutide and subcutaneous GLP-1RAs

There is currently insufficient clinical experience in switching between oral semaglutide and subcutaneous GLP-1RAs. The recommendation is to adhere to the posology described within the labeling of each agent when switching. However, the product labeling for oral semaglutide provides guidance for switching between the oral and subcutaneous formulations of semaglutide. Patients receiving oral semaglutide 14 mg can transition to subcutaneous semaglutide 0.5 mg once weekly on the day after the last oral dose.^13^ Those treated with subcutaneous semaglutide 0.5 mg can switch to oral semaglutide 7 or 14 mg up to 7 days after the last subcutaneous dose.^13,23^

## Clinical efficacy of oral semaglutide

In order to counsel patients on the therapeutic response they can anticipate when receiving oral semaglutide treatment, it is important that pharmacists are familiar with the key results from the PIONEER clinical trial program.^24^ The main PIONEER program comprised 8 global randomized controlled trials (PIONEER trials 1-8) of at least 26 weeks’ duration.^25-32^ PIONEER trials 1 through 5, 7, and 8 included patients on a range of background treatment regimens (including diet and exercise, oral glucose-lowering agents, and insulin) and involved a number of different comparators: placebo; empagliflozin (an SGLT2 inhibitor); sitagliptin (a dipeptidyl peptidase-4 [DPP-4] inhibitor); and liraglutide (a GLP-1RA). Furthermore, the PIONEER 5 trial specifically evaluated oral semaglutide 14 mg in patients with renal impairment,^27^ while the PIONEER 7 trial assessed a flexible dosing regimen of oral semaglutide.^28^ A CV outcomes trial (CVOT), the PIONEER 6 trial, was also included in the PIONEER program and is discussed later in this article.^26^ Two further trials (PIONEER trials 9 and 10) were conducted in Japanese subjects, comparing oral semaglutide with the GLP-1RAs liraglutide and dulaglutide.^33,34^

In brief, results from PIONEER trials 1 through 5 and 8 demonstrated the efficacy of oral semaglutide in improving glycemic control and reducing body weight when the dosing and administration instructions described previously were followed.^25,27,29-32^ For patients whose data were analyzed regardless of study drug discontinuation or rescue medication use, estimated mean HbA_1c_ reductions from baseline at week 26 ranged from 0.9% to 1.2% with use of oral semaglutide 7 mg and from 1.0% to 1.4% with use of oral semaglutide 14 mg.^25,27,29-32^ Estimated mean body weight reductions from baseline at week 26 ranged from 2.2 to 2.4 kg with use of oral semaglutide 7 mg and from 3.1 to 4.4 kg with use of oral semaglutide 14 mg.^25,27,29-32^ With flexible dose adjustment in PIONEER 7, estimated mean HbA_1c_ changes from baseline at week 52 were –1.3% with use of oral semaglutide and –0.8% with use of sitagliptin, resulting in an ETD of –0.5% (95% confidence interval [CI], –0.7% to –0.4%); *P* < 0.0001).^28^

The PIONEER 4 trial was a global head-to-head trial comparing oral semaglutide 14 mg with the injectable GLP-1RA liraglutide (1.8 mg) or placebo in patients on metformin with or without an SGLT2 inhibitor. Oral semaglutide 14 mg was noninferior to subcutaneous liraglutide 1.8 mg in decreasing HbA_1c_ from baseline at week 26 (ETD for primary endpoint, –0.1% [95% CI, –0.3% to 0.0%]; *P* < 0.0001 for noninferiority), and reductions were significantly in favor of oral semaglutide at week 52 (ETD, –0.3% [95% CI, –0.5% to –0.1%]; *P* = 0.0002).^29^ Furthermore, oral semaglutide provided superior weight loss vs liraglutide at week 26 (ETD for confirmatory secondary endpoint, –1.2 kg [95% CI, –1.9 kg to –0.6 kg]; *P* = 0.0003), and a statistically significant difference favoring oral semaglutide was maintained at week 52.^29^ These findings were supported by results of the Japanese trials. In the PIONEER 9 trial, patients who received oral semaglutide 14 mg had a significantly greater mean reduction in HbA_1c_ than those who received liraglutide 0.9 mg once daily at 26 weeks (ETD, –0.4% [95% CI, –0.7% to –0.1%]; *P* = 0.0077) and a significantly greater mean reduction in body weight at week 52 (ETD, –2.7 kg [95% CI, –3.8 kg to –1.5 kg]; *P* < 0.0001).^34^ Moreover, in the PIONEER 10 trial, the estimated mean HbA_1c_ and body weight changes from baseline at week 52 (these were supportive secondary efficacy endpoints) were –1.7% and –1.6 kg with oral semaglutide 14 mg and –1.4% and 1.0 kg with dulaglutide 0.75 mg once weekly, respectively (*P* = 0.0170 for HbA_1c_ change and *P* < 0.0001 for body weight change, with both comparisons favoring oral semaglutide).^33^

A population pharmacokinetics (PK) analysis was performed to assess whether the route of administration (oral vs subcutaneous) affected efficacy and GI tolerability response in relation to semaglutide exposure.^35^ Data were analyzed across a series of clinical trials in the SUSTAIN program for once-weekly subcutaneous semaglutide and the PIONEER program for once-daily oral semaglutide, and the results showed that subcutaneous and oral semaglutide had similar exposure-response relationships for efficacy and tolerability. In addition, although there was greater variability in plasma concentrations for oral semaglutide, this did not affect the response.

## Safety profile of oral semaglutide

Pharmacists play an important role in ensuring that patients understand the most common AEs that they may experience, and how to manage these AEs, when initiating any new treatment. Across PIONEER trials 1 through 5, 7, and 8, the safety and tolerability of oral semaglutide were found to be consistent with those for the injectable GLP-1RA class, with GI-related AEs being the most common.^25,27-32^ In the PIONEER 1 trial, which evaluated oral semaglutide vs placebo in patients treated with diet and exercise, rates of any AE were 58%, 53%, and 57% with use of oral semaglutide 3, 7, and 14 mg, respectively, and 56% with placebo use.^25^ The corresponding incidence rates of serious AEs were 3%, 2%, and 1% with oral semaglutide 3, 7, and 14 mg, respectively, and 4% with placebo use.^25^ In PIONEER 4, at least 1 AE occurred in 80% of patients treated with oral semaglutide 14 mg, 74% of those treated with liraglutide 1.8 mg, and 67% of placebo recipients.^29^ Serious AEs occurred at rates of 11% with use of oral semaglutide 14 mg, 8% with use of liraglutide 1.8 mg, and 11% with placebo use.^29^

Although GI AEs, typically nausea, diarrhea, and vomiting, were the most common AEs across the PIONEER program, the majority of patients did not experience GI AEs.^25,27-32^ For example, in the PIONEER 1 trial the incidence rates of nausea/diarrhea/vomiting were 8%/9%/3%, 5%/5%/5%, and 16%/5%/7% with oral semaglutide 3, 7, and 14 mg, respectively, and 6%/2%/2% with placebo use.^25^ Furthermore, in the head-to-head PIONEER trial 4, nausea/diarrhea/vomiting occurred in 20%/15%/9% of patients receiving oral semaglutide 14 mg, 18%/11%/5% receiving liraglutide 1.8 mg, and 4%/8%/2% receiving placebo.^29^ It is also important to note that the GI AEs tended to be mild to moderate in severity and transient in nature, with few discontinuations of study drug across the trial program.^25-32^ The incidence rates of premature study drug discontinuation due to GI AEs in PIONEER 1 were 2%, 2%, and 5% with use of oral semaglutide 3, 7, and 14 mg, respectively, and 1% with placebo use.^25^ In the PIONEER 4 trial, the rates of premature study drug discontinuation due to GI AEs were 8% with oral semaglutide 14 mg, 6% with liraglutide 1.8 mg, and 2% with placebo use.^29^ Moreover, the PK analysis mentioned in the previous section also showed that the route of administration (oral vs subcutaneous) did not affect GI tolerability response vs semaglutide exposure.^35^

The incidence rates of diabetic retinopathy complications reported in the PIONEER program were similar for patients treated with oral semaglutide, placebo recipients, and patients who received active comparators. The majority of cases were identified during routine eye examinations and did not require further treatment. If oral semaglutide is used in patients with a history of diabetic retinopathy, they should be monitored for progression of diabetic retinopathy complications,^13^ consistent with current standard care for patients with diabetes.

Patients should be advised of the potential risk of thyroid C-cell tumors, including medullary thyroid carcinoma (MTC), as indicated in the boxed warning in the label for oral semaglutide and other long-acting subcutaneous GLP-1RAs.^8-11,13^ Consequently, oral semaglutide is contraindicated in patients with a personal or family history of MTC and those with multiple endocrine neoplasia syndrome type 2 (MEN 2).

Patients experiencing signs and symptoms of pancreatitis (severe abdominal pain that may radiate to the back and is sometimes accompanied by vomiting) should discontinue use of oral semaglutide and consult their physician.^13^ A small number of cases of acute pancreatitis were reported in the PIONEER trials, with similar rates in patients treated with oral semaglutide and those treated with comparator agents.^25,27-32^ However, a recent meta-analysis suggested the GLP-1RAs are not associated with a change in the risk of pancreatitis.^36^

The PIONEER clinical trials showed that adding oral semaglutide to an existing treatment regimen was associated with a low risk of hypoglycemia, similar to that reported for empagliflozin, sitagliptin, and liraglutide and consistent with that for the GLP-1RA drug class as a whole.^13,25,27-32^

### Cardiovascular safety profile.

ASCVD is the leading cause of death in patients with T2D^37^; therefore, it is important to consider the CV safety profile of new medications for T2D. More specifically, regulatory guidelines stipulate that a premarketing evaluation of CV safety should exclude an 80% excess in CV risk compared with placebo use and that an estimated increased risk of 30% to 80% would warrant a postmarketing safety trial.^38^ Following a 2-year CVOT, subcutaneous semaglutide was recently approved by FDA for use in reducing the risk of major adverse CV events (CV death, nonfatal stroke, or nonfatal myocardial infarction [MI]) in adults with T2D and established CV disease.^9,39^ The PIONEER 6 trial was a placebo-controlled CVOT that was designed to rule out an 80% excess in CV risk with use of oral semaglutide among patients with T2D at high CV risk (defined as age of ≥50 years with established CVD or CKD or age of ≥60 years with CV risk factors only).^26,40^ A total of 3,183 patients were randomly assigned to receive oral semaglutide 14 mg (*n =* 1,591) or a placebo (*n =* 1,592), both given in addition to standard of care for T2D and CVD or CKD. The median follow-up time in the trial was 15.9 months (range, 0.4-20 months). The primary outcome (first occurrence of a major adverse CV event [death from CV causes, nonfatal MI, or nonfatal stroke]) occurred in 3.8% of patients receiving oral semaglutide and 4.8% receiving placebo. The trial met its primary objective, with oral semaglutide found to be noninferior to placebo for the 3-point major adverse CV event primary endpoint (hazard ratio [HR], 0.79; 95% CI, 0.57, to 1.11; *P* < 0.001 for noninferiority and *P =* 0.17 for superiority). Results for the components of the primary outcome showed that death from CV causes occurred in 0.9% of patients in the oral semaglutide group and 1.9% in the placebo group (HR, 0.49; 95% CI, 0.27-0.92). First nonfatal MI events were experienced by 2.3% and 1.9% of patients receiving oral semaglutide 14 mg and placebo, respectively (HR, 1.18; 95% CI, 0.73-1.90). First nonfatal stroke events were reported in 0.8% and 1.0% of patients receiving oral semaglutide 14 mg and placebo, respectively (HR, 0.74; 95% CI, 0.35-1.57). These results confirmed the absence of unacceptable excess CV risk with use of oral semaglutide, a finding that was consistent with results of several other GLP-1RA CVOTs.^39,41-45^ Analyses of neither the primary endpoint nor its components were powered to show a difference between treatments. A larger and longer CVOT powered to assess a potential CV benefit with oral semaglutide use is ongoing (ClinicalTrials.gov identifier, NCT03914326).

Managing patient expectations of adverse events.To help manage patient expectations and optimize treatment adherence, pharmacists should ensure that patients initiated on treatment with oral semaglutide are aware that while they may experience GI AEs, such events are usually mild to moderate in severity, are generally temporary, and often occur during dose escalation.^25,27-32^ As GI AEs are typically more frequent with increasing dose and faster dose escalation,^13,22^ it is important that the recommended dose-titration strategy (Figure 2) is followed in order to mitigate the likelihood of GI AEs occurring.^13^ Treatment initiation at a low dose before escalation is similarly recommended for injectable GLP-1RAs.^9-11^ Furthermore, there are practical strategies for effective management and mitigation of GI AEs that can be employed.

We suggest that if a patient experiences GI AEs despite the recommended dose-titration strategy, individualized dose titration and/or dose adjustments could be considered to mitigate AEs and help maintain adherence to treatment. More specifically, patients could be maintained on the lowest standard dose (3 mg) for up to 8 weeks until GI AEs have subsided. In situations when GI AEs are more than mild in severity, the current dose could be temporarily maintained or decreased until the severity of symptoms is reduced. This guidance is given in light of the flexible dose-adjustment approach successfully used in the PIONEER 7 study.^28^ In that trial, patients receiving oral semaglutide were initiated at a 3-mg dose, which was maintained until week 8. At week 8, patients were escalated to a 7-mg dose if tolerability was acceptable, and this occurred in 73% of patients. Subsequently, the dose was adjusted every 8 weeks based on HbA_1c_ level and GI tolerability. Where the HbA_1c_ concentration was 7.0% or higher, the current dose of oral semaglutide was escalated to the next dose level. If patients reported moderate to severe nausea or vomiting for 3 or more days in the week prior to the scheduled visit, the dose was maintained or decreased to a minimum of 3 mg once daily, irrespective of the HbA_1c_ level and at the investigator’s discretion. At week 52, 19 (9%), 64 (30%), and 126 (59%) of the patients still on treatment were receiving 3-, 7-, and 14-mg doses of oral semaglutide, respectively. Flexible dose adjustment enabled individualized tolerability management while still providing significantly greater glycemic control and decreases in body weight than have been reported with use of the DPP-4 inhibitor sitagliptin. Overall total treatment satisfaction, treatment convenience, and flexibility were similar for once-daily oral semaglutide and once-daily oral sitagliptin. These results could be interpreted to indicate that there was little impact of oral semaglutide dosing conditions on treatment convenience or satisfaction.^28^

We would suggest advising patients that GLP-1RAs increase satiety and they may feel full more quickly during treatment with oral semaglutide. Patients should be counseled to stop eating when they feel full, as overeating may be a prelude to vomiting associated with GLP-1RA therapy. It has been reported that patients feel significantly fuller after a standardized fat-rich breakfast meal with use of oral semaglutide vs placebo.^46^ It is important to also note that satiety has been shown to be increased during treatment with oral semaglutide vs placebo, and this did not appear to be related to an increased aversion to food.^46^ General advice for patients treated with GLP-1RAs is to eat smaller meals, particularly for the first few weeks, which could help minimize nausea.^47-49^ Moreover, patients may refrain from consuming greasy food and try to eat food that is easy to digest.^47-49^ The key points to communicate to patients with regard to GI AEs are summarized in Box 1.

**Box 1. Key Take-Home Messages and Counseling Tips for Optimal Management of Patients With Type 2 Diabetes**

**Dosing recommendations** ^13^

- Oral semaglutide should be taken on an empty stomach when the patient first wakes up.
- Patients should take oral semaglutide with no more than a sip (up to 120 mL, or 4 fluid ounces) of plain water.
- Patients should wait at least 30 minutes after taking oral semaglutide before consuming any food, other drinks, or other medications.
- It is best to eat within 30 to 60 minutes after taking oral semaglutide.
- Oral semaglutide tablets must be kept in the blister card until use and therefore should not be placed in a pill box.

**Managing patient expectations regarding glycemic efficacy** ^13,25,27,29-32^

- Oral semaglutide 7 mg is effective at lowering glycated hemoglobin (HbA_1c_), with estimated mean HbA_1c_ reductions from baseline of approximately 0.9% to 1.2% after 26 weeks; patients may expect HbA_1c_ reductions of this magnitude, though actual reductions in HbA_1c_ will vary between patients.
- If further HbA_1c_ reductions are required, the patient can escalate the dose of oral semaglutide from 7 mg to 14 mg. HbA_1c_ reductions of approximately 1.0% to 1.4% can be expected with oral semaglutide 14 mg after 26 weeks, but as above, this can vary between patients.
- Oral semaglutide 3 mg is intended to be used when initiating treatment, to help patients become accustomed to taking a glucagon-like peptide 1 receptor agonist and to mitigate gastrointestinal adverse events (GI AEs) before patients are dose-escalated to oral semaglutide 7 mg; it will not provide the full HbA_1c_-lowering effect that is achievable with oral semaglutide and is not intended to be the final maintenance dose

**Managing patient expectations regarding body weight** ^25,27,29-32^

- Patients may lose an average of up to 2.2 to 2.4 kg after 26 weeks of treatment with oral semaglutide 7 mg, and up to 3.1 to 4.4 kg with oral semaglutide 14 mg; as these are study averages, actual results will vary between patients, as well as their adherence to lifestyle changes.
- Patients should be encouraged to stop eating upon reaching satiety.

**Comorbidities** ^13,27^

- Oral semaglutide has a favorable safety profile and is effective for patients with moderate renal impairment.
- Patients with renal impairment should be advised to take precautions to avoid fluid depletion and dehydration when experiencing GI AEs, as this may adversely affect renal function, and if they experience severe or prolonged GI AEs, they should be advised to speak with their healthcare provider.
- No dose adjustment of oral semaglutide is recommended in patients with renal impairment.
- No dose adjustment of oral semaglutide is recommended in patients with hepatic impairment.

**Concomitant medications** ^13,57-60^

- It is very important to follow the dosing conditions for oral semaglutide to ensure that other medications the patient may be using do not affect the efficacy of oral semaglutide, or vice versa.
- Oral semaglutide does not affect exposure of lisinopril, warfarin, digoxin, or ethinylestradiol/levonorgestrel.
- Oral semaglutide increases exposure of metformin, furosemide, and rosuvastatin; however, increases in exposure are not considered to be clinically relevant.
- Oral semaglutide increases exposure of levothyroxine.
- It is recommended that increased clinical or laboratory monitoring should be considered for medications that require clinical monitoring or those that have a narrow therapeutic index (eg, warfarin and levothyroxine).

**Management of AEs** ^13,25,27-32,47-49^

- Patients should be informed that GI AEs can occur after initiating treatment with or escalating the dose of oral semaglutide, but that these events are generally mild-to-moderate and transient in nature.
- The dose-escalation strategy and flexible dose adjustment can be employed effectively during treatment to enable individualized tolerability management while still providing effective glycemic control and decreases in body weight.
- To help manage expectations, it is important to highlight to patients that hunger is likely to be satiated more quickly than they are accustomed to when they start taking oral semaglutide.
- To help prevent or manage any GI AEs that may occur, patients should be advised to eat smaller meals for the first few weeks and stop eating when they feel full, to refrain from consuming greasy food, and to try to eat food that is easy to digest.

## Oral semaglutide use in patients with renal or hepatic impairment and/or upper GI disease

The potential impact of renal impairment, a common comorbidity in patients with T2D,^50^ on the PK of oral semaglutide (5 mg for 5 days, followed by 10 mg for 5 days) has been assessed in a multicenter, open-label, multiple-dose phase 1 trial.^51^ Creatinine clearance (CL_cr_) was determined by the Cockcroft-Gault formula, and subjects with normal renal function (*n* = 24), mild renal impairment (CL_cr_ of 60-89 mL/min/1.73 m^2^; *n* = 12), moderate renal impairment (CL_cr_ of 30-59 mL/min/1.73 m^2^; *n* = 12), severe renal impairment (CL_cr_ of 15-29 mL/min/1.73 m^2^; *n* = 12), or end-stage renal disease (ESRD) requiring hemodialysis (*n* = 11) were included; 12 subjects had T2D. No consistent variation or clinically relevant pattern of increase or decrease in semaglutide exposure was identified in comparing subjects with renal impairment of varying levels of severity and those with normal renal function.^51^ While the proportion of patients experiencing AEs was higher among those with renal impairment (mild, 58%; moderate, 58%; severe, 25%; ESRD, 27%) than those with normal renal function (21%), the severity of renal impairment did not appear to impact the rate of AEs.^51^ Therefore, no oral semaglutide dose adjustment is recommended for patients with renal impairment.^13^ However, precautions should be taken to avoid fluid depletion and dehydration if patients experience GI AEs, as this may adversely affect renal function.^13^ The PIONEER 5 trial showed that oral semaglutide was superior to placebo in decreasing HbA_1c_ (ETD, –0.8%; *P* < 0.0001) and body weight (ETD, –2.5 kg; *P* < 0.0001) in patients with T2D and moderate renal impairment (defined as an estimated glomerular filtration rate of 30-59 mL/min per 1.73 m^2^).^27^ No unexpected safety findings were reported, and the AE profile was consistent with that for the GLP-1RA class and that for a T2D population with moderate renal impairment. The efficacy and safety of oral semaglutide in patients with severe renal impairment or ESRD have not been assessed in clinical trials. Data suggest the GLP-1RA class may have a renoprotective effect in patients with moderate or severe renal function.^45,52-54^ In the LEADER study, liraglutide use was associated with a slower deterioration in renal function than that seen with placebo use.^54^ In the REWIND study, treatment with dulaglutide led to a lesser decrease in renal function than occurred with placebo use.^45^ Oral semaglutide was shown to decrease the urinary albumin-to-creatinine ratio, a risk marker for kidney damage, similar to findings with liraglutide and subcutaneous semaglutide.^27,53^

Hepatic impairment is also a potential comorbidity in patients with T2D, and the impact of hepatic impairment on the PK of oral semaglutide (5 mg for 5 days, followed by 10 mg for 5 days) has been investigated in a multicenter, open-label, multiple-dose phase 1 trial.^55^ Subjects with normal hepatic function (*n =* 24), or mild (*n =* 12), moderate (*n =* 12), or severe (*n =* 8) hepatic impairment, as determined by Child-Pugh score, were included; 6 subjects had T2D. Similar semaglutide exposure was observed across the hepatic function groups, with no apparent effect of hepatic impairment on semaglutide PK. Reported AEs were in line with those for other GLP-1RAs, and no safety concerns were identified.^55^ Therefore, no oral semaglutide dose adjustment is recommended for patients with hepatic impairment.^13^

As the stomach is the key site of absorption of oral semaglutide, the effect of upper GI disease on the PK of oral semaglutide was evaluated in an open-label, parallel-group trial.^56^ Patients with T2D and upper GI disease (chronic gastritis [*n =* 5], gastroesophageal reflux disease [*n =* 8], or both [*n =* 23]) or without upper GI disease (*n =* 19) were included.^56^ No statistically significant difference in semaglutide exposure was observed in patients with vs patients without upper GI disease.^56^ Therefore, no dose adjustment is recommended.^13^ Caution would still be prudent in patients with established GI disease due to the AE profile of oral semaglutide and the GLP-1RA class as a whole. However, no safety concerns were identified by this study,^56^ and the most common AEs observed were generally in line with those observed with use of other GLP-1RAs.^1,7-11^

## Concomitant medication use with oral semaglutide

**Figure 3.**
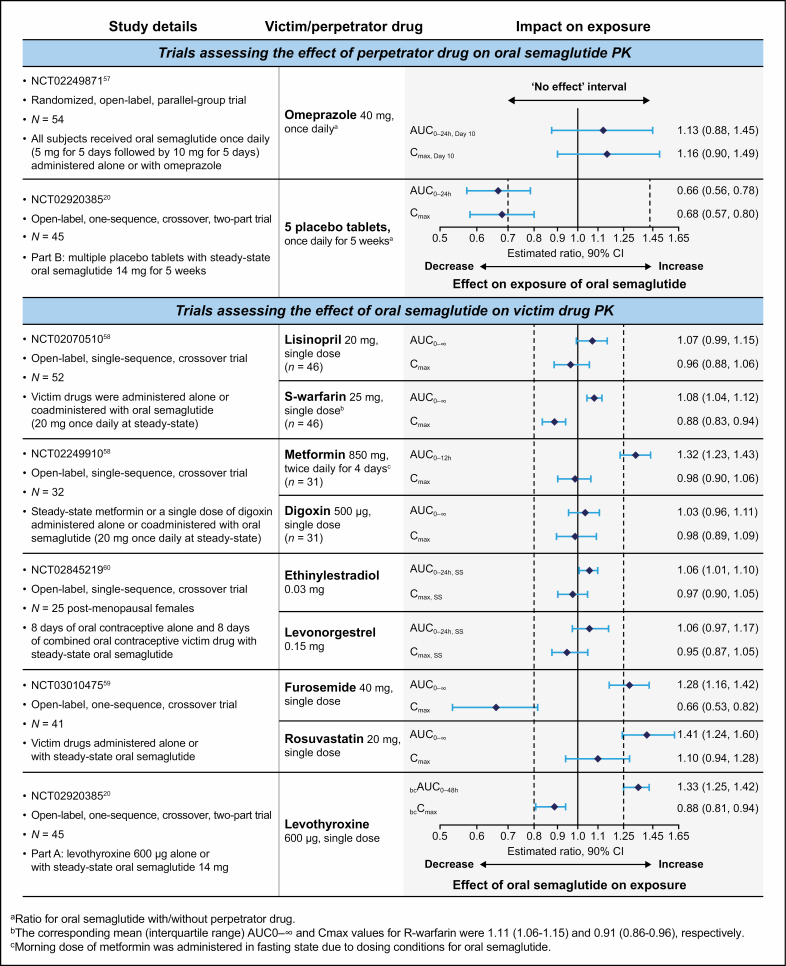
Key phase 1 drug-drug interaction information for coadministration with oral semaglutide. All study participants were healthy adult subjects. AUC indicates area under the curve; bc, baseline-corrected; CI, confidence interval; *C*_max_, maximum serum concentration; PK, pharmacokinetics; SS, steady state. AUC and *C*_max_ ratios are for victim drug with/without oral semaglutide unless otherwise stated.

Many patients with T2D require a complex medication regimen to manage T2D and/or other chronic comorbid conditions (eg, hypertension, dyslipidemia, CKD, CV disease).^37,50^ It is therefore important to understand if the PK of these medications may be affected by oral semaglutide, or vice versa. In particular, it is possible that the known effect of GLP-1RAs in delaying gastric emptying may impact the absorption of other oral medications.^13^ The effect on the PK of oral semaglutide when the drug is administered in combination with omeprazole or multiple oral placebo tablets has been assessed (Figure 3).^20,57^ Semaglutide exposure appeared slightly increased when oral semaglutide was administered with omeprazole vs alone; however, the differences were not statistically significant, and concomitant omeprazole administration did not affect semaglutide *t*_max_ or t_½_.^57^ It is speculated that this slight increase in semaglutide exposure may be due to the proton pump inhibitor–associated increase in gastric pH influencing semaglutide absorption.^57^ Absorption of oral semaglutide has been shown to decrease when coadministered with multiple (ie, 5) placebo tablets.^20^ This phenomenon is addressed in the clinical dosing guidance for oral semaglutide, in that patients should be advised to wait at least 30 minutes before taking other oral medications (Figure 3).^13^

Drug interaction studies also investigated whether coadministration with oral semaglutide would affect the PK of lisinopril, warfarin, digoxin, or metformin^58^; furosemide or rosuvastatin^59^; or the combined oral contraceptive ethinylestradiol/levonorgestrel (Figure 3).^60^ Oral semaglutide (a single dose) did not impact exposure to lisinopril, warfarin, digoxin, or ethinylestradiol/levonorgestrel.^58,60^ The International Normalized Ratio (INR) from time 0 to 144 hours after dosing (INR_max_) for warfarin was also not affected (the estimated ratio [with vs without oral semaglutide] was 0.98; 90% CI, 0.96-1.01).^58^ Although oral semaglutide had a small impact on metformin, furosemide, and rosuvastatin exposure, the changes observed were not considered clinically relevant, and no adjustment of the dose of these drugs is likely to be required in clinical practice (Figures 2 and 3).^13,58,59^ The observed changes in drug exposure may be due to the known effect of GLP-1 analogs in delaying gastric emptying.^61^

Oral semaglutide and levothyroxine have similarities in their dosing requirements,^13,62^ and when coadministered with oral semaglutide at steady state, total thyroxine exposure after a single 600-µg dose has been shown to increase by 33% without affecting maximum exposure (Figure 3).^20^ Therefore, it is important to follow the dose administration instructions for oral semaglutide closely, monitor thyroid parameters, and adjust treatment if required.^13^ Patients should be counseled to speak to their pharmacist or healthcare provider as soon as possible if any unexpected change in health is noticed. Consideration could be given to taking levothyroxine at bedtime, at least 3 hours after the evening meal.^63^ However, it should be noted that none of the studies investigating bedtime dosing of levothyroxine included coadministration of oral semaglutide.

In summary, the safety profile of oral semaglutide was as expected for agents in the GLP-1RA drug class across all of the drug interaction studies. The key information regarding use of concomitant medications with oral semaglutide for pharmacists to be aware of is outlined in Figure 2 and summarized in Figure 3.

## Conclusion

Approval of oral semaglutide, the first oral GLP-1RA, has provided patients with a new, potentially more convenient treatment option that offers similar or improved efficacy relative to injectable GLP-1RAs and tolerability consistent with that of other agents in the GLP-IRA class. Although oral semaglutide is a recently available treatment, some analyses suggest that oral semaglutide could offer a potentially cost-effective treatment relative to other agents, including GLP-1RAs and the SGLT2 inhibitor empagliflozin.^64-66^ It is crucial, however, that pharmacists work closely with patients to ensure their understanding of the correct dosing instructions for oral semaglutide: (1) take on an empty stomach when first awake, with 4 ounces or less of water; (2) swallow whole (do not chew, split, or crush); and (3) wait at least 30 minutes prior to taking food, other beverages, or other oral medications.^13^ In addition, patients should be aware of potential GI AEs, reassured that these effects are typically mild to moderate and transient in nature, and that a dose-titration schedule can help manage GI AEs. Patients should also be informed that changes to their eating behavior, such as eating smaller meals, minimizing overeating, and avoiding greasy food, will also help minimize GI AEs. These approaches will help pharmacists provide patients with the support and counseling they need to successfully navigate the initiation and continuation of oral semaglutide therapy.

## Disclosures

Development of this article, including assistance with medical writing and editorial support, was supported by Novo Nordisk Inc., which was provided with the opportunity to perform a medical accuracy review. Dr. Kane is on the speakers bureaus of Amarin Pharmaceuticals and Novo Nordisk, and is the recipient of an investigator-initiated research grant from AstraZeneca. Dr. Triplitt is on the speakers bureaus of AstraZeneca and Janssen and is a consultant for Novo Nordisk, Eli Lilly, and Xeris. Dr. Solis-Herrera has served as a consultant for Sanofi/Lexicon.
