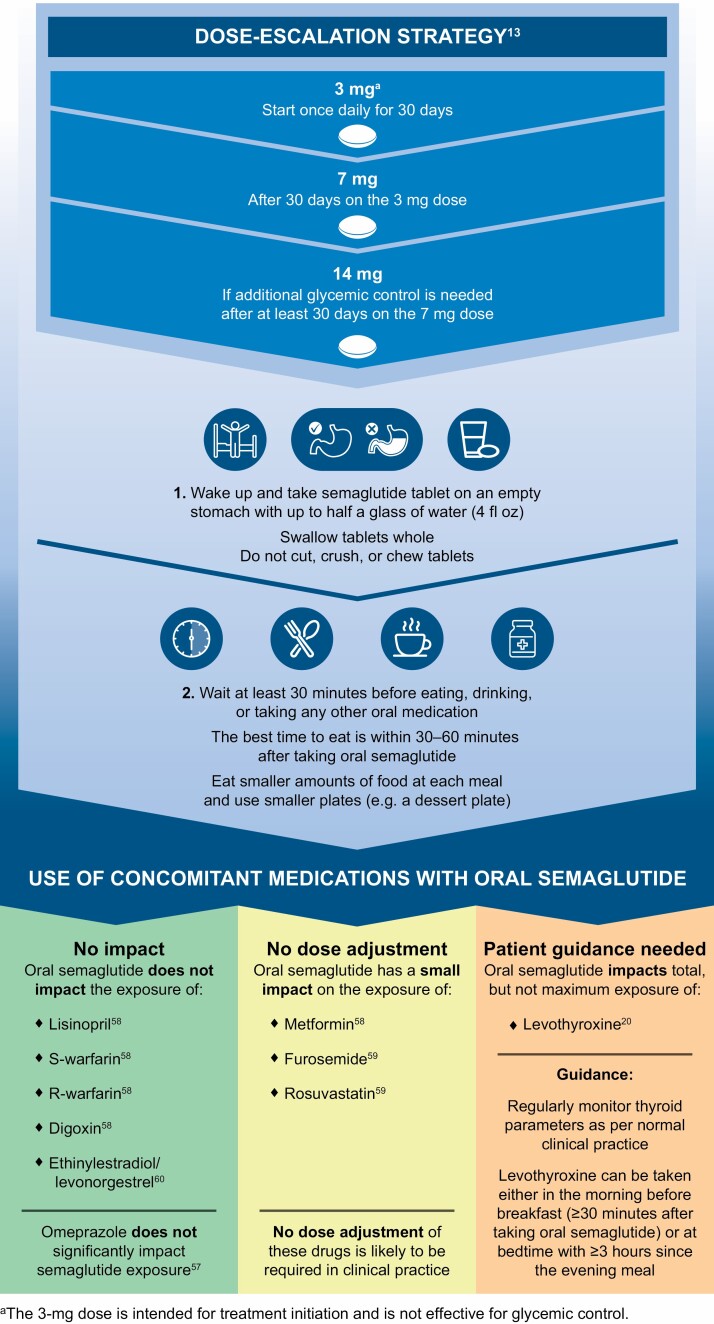# Correction Notice

**DOI:** 10.1093/ajhp/zxab022

**Published:** 2021-02-16

**Management of type 2 diabetes with oral semaglutide: Practical guidance for pharmacists,** Michael P. Kane, Curtis L. Triplitt, Carolina D. Solis-Herrera. *Am J Health-Syst Pharm*. 2020 (Clinical Review).

In the originally published version of this manuscript, several errors were noted and are listed in this corrigendum.

Errors related to the following reference numbers were noted in Figure 2, Box 1, and on page 7: 28, 45, 52, 52, 54, 55, 59, 60.

The term “oral” was inadvertently omitted from the following text: In the “Key Points” section, “Patients treated with semaglutide should be counselled about optimal dosing” should read: “Patients treated with oral semaglutide should be counselled about optimal dosing”. In the “Oral semaglutide use in patients with renal or hepatic impairment and/or upper GI disease” section, “Therefore, no semaglutide dose adjustment is recommended for patients with renal impairment” should read: “Therefore, no oral semaglutide dose adjustment is recommended for patients with renal impairment”. In addition, “Therefore, no semaglutide dose adjustment is recommended for patients with hepatic impairment” should read “Therefore, no oral semaglutide dose adjustment is recommended for patients with hepatic impairment”.

Under the “Managing patient expectations of adverse events“ heading, the following sentence should read: “The median follow-up time in the trial was 15.9 months (range, 0.4–20 months).”.

Reference 23 should read: “Almandoz JP, Lingvay I, Morales J, et al. Switching between glucagon-like peptide- 1 receptor agonists: rationale and practical guidance. *Clin Diabetes*. 2020;38:390–402” instead of “Almandoz JP, Lingvay I, Morales J, et al. Switching between glucagon-like peptide- 1 receptor agonists: rationale and practical guidance. *Clin Diabetes*”.

Reference 24 should read: “Thethi TK, Pratley R, Meier JJ et al. Efficacy, safety and cardiovascular outcomes of once-daily oral semaglutide in patients with type 2 diabetes: the PIONEER Programme. *Diabetes Obes Metab*. 2020;22:1263–1277.” instead of “Thethi TK, Pratley R, Meier JJ et al. Efficacy, safety and cardiovascular outcomes of once-daily oral semaglutide in patients with type 2 diabetes: the PIONEER Programme”.

These have now been corrected online.

DOI 10.1093/ajhp/zxaa413